# Ophthalmic Conditions in the Government Schools in Egypt

**Published:** 1909-06-12

**Authors:** 


### SPECIAL ARTICLE.

### OPHTHALMIC CONDITIONS IN THE GOVERNMENT SCHOOLS IN EGYPT.

A recently published monograph endeavours to show briefly the methods adopted by the Government to stamp out, or at all events relieve, the ophthalmic distress under which the inhabitants live. Egypt is always looked upon as the home of trachoma; hence by far the greater part of the work undertaken and the treatment adopted is in an effort to combat this disease, and to relieve the sufferers already afflicted by it. The Director-General of the Department of Public Health was asked how this could be best carried out, and he recommended the establishment of a travelling ophthalmic hospital.

The duties of this hospital were to treat cases from the neighbourhood surrounding the temporary resting-place of the camp-hospital, and to train the Egyptian doctors attached to the hospital in modern ophthalmic surgery.

The treatment of trachoma on a moderately large scale is an exceedingly difficult and lengthy business, unless the patients can be collected together, placed under the most suitable hygienic conditions having regard to the extremely contagious nature of the disease, and treated every day by skilled hands; but when an endeavour is made to treat a whole nation, especially one not noted for habits of cleanliness (largely due to the insufficient water supplies), one can only be surprised that any advance at all has been made. It was very early found that to treat the aged for a short time, the inhabitants of some town and its outlying neighbourhood for a few months during the year, and to repeat this year after year, though necessitating the greatest amount of hard work, was barely touching the evil, so attempts have been made to treat the young and growing generation by means of school inspections and treatment of any afflicted scholars. The young subjects react more readily to treatment, and do not present the deformities due to long continuance of the disease which make many cases almost untreatable. Together with actual treatment as much as possible is done to educate the school children in habits of cleanliness, and in taking precautions not to become infected themselves or infect anyone else.

Part I. of the report contains a *résumé* of the work done in the year by the two travelling hospitals now established. The number of new patients treated has been 7,446; the average number of attendances of patients regularly treated, 19; 3,173 patients were seen who were incurable, and were sent away after the first examination. This shows the enormous difficulties met with in treating a whole nation, since large numbers of those "incurable" remain very active carriers of infection. Over 2,000 persons were seen who had been operated upon for trichiasis by charlatans, and in most cases were worse than before, further increasing the difficulties and rendering it more than ever necessary to train the Egyptian doctor in ophthalmic practice.

Part II. deals with the means of education in Egypt; the cause of the prevalence of eye disease in Egypt and its nature, including some observations on the stages of trachoma; and concludes with "Facilities for Ophthalmic Treatment in Egypt," the gist of the latter being a somewhat detailed account of the extreme difficulties met with and to to be overcome before much progress towards the relief of the large numbers afflicted can be effected. Under cause and prevalence, a remark is made "that it is comparatively rare to find anyone above the age of twenty years who exhibits no trace either of active or passive trachoma." The writer divides the stages of trachoma into four. The first includes the disease in its earliest stages; the second the very active, well-developed granulation stage; the third the stage of cicatrisation; and the fourth the condition in which there is a smooth conjunctiva seamed by white lines of connective tissue, the stage of cured trachoma. By "cured" trachoma is evidently meant the condition when the activity of the disease has come to an end; but slow contraction of conjunctiva causing deformities of the lids is still going on and continues to do so for many years, though the disease is in all probability non-infective.

Part III. deals with statistics relative to numbers of cases seen in the schools and at different school years. In this is mentioned, "Out of 485 pupils in the school at the beginning of the school year, only 21 were found to be absolutely healthy (as regards their eyes), 464, or 95.67 per cent., being infected with trachoma." Also 15 of those at first classed as healthy developed trachoma during the school year, not necessarily, of course, infected at school or within school hours, but much more likely to have acquired it at home. It was also found that there was a greater percentage of trachoma cases amongst those who had bad vision than amongst those who had normal or good vision. Amongst healthy patients in England various forms of conjunctivitis are found more frequently amongst those with some defect in vision than amongst the absolutely normal sighted.

Part IV. deals with the means adopted at one of the largest Government schools (Tantah) with a view to the improvement of the ophthalmic conditions. These resolve themselves into detailed examination of the eyes and sight of every pupil at the beginning and end of the school year, for the compilation of statistics, etc. Prophylactic treatment for all pupils with severe trachoma, apparently within school hours. Still further treatment of those afflicted, carried out at request of the pupil and out of school hours. The atropinisation and refraction of all scholars with defective vision. Information given to guardians, parents, etc., when those scholars in their charge have such a diseased condition of the eyes that operative treatment is considered necessary. Power given to the head master to send any pupil who develops a discharge from his eyes to the ophthalmic surgeon.

Part V. deals with the results of treatment. The complete examinations of all pupils were made, one at the beginning, the other at the end of the year, and the results tabulated. Although prophylactic treatment (which consisted in the instillation of antiseptic drops into the eyes of all suffering from severe trachoma) was carried out daily for the 211 pupils with severe trachoma, the writer considers this has but little effect on the disease, and includes them among the statistics as being untreated, and states that of the treated cases 87.2 per cent. showed improvement at the end of the school year, while of the untreated cases only 5.9 per cent. showed improvement. In the ordering of glasses for those requiring them extreme difficulty was found in many cases to get frames to fit, owing to the abnormal length of the eyelashes, often reaching eleven millimetres, which necessitates the adjustment of the lenses at a considerable distance from the eyes.

The whole report, which has the advantage of being written by "the man on the spot," shows that much has been done both in the way of treatment and in organisation towards the effective treatment of a nation which for centuries has been afflicted by one of the most destructive forms of eye disease, and which it will take generations to cure.

